# Apoptotic-Induced Effects of *Acacia Catechu* Willd. Extract in Human Colon Cancer Cells

**DOI:** 10.3390/ijms21062102

**Published:** 2020-03-19

**Authors:** Elda Chiaino, Matteo Micucci, Miriam Durante, Roberta Budriesi, Roberto Gotti, Carla Marzetti, Alberto Chiarini, Maria Frosini

**Affiliations:** 1 Dipartimento di Scienze della Vita, Università di Siena, Via Aldo Moro 2, 53100 Siena, Italy; chiaino@student.unisi.it (E.C.); durante6@unisi.it (M.D.); 2 Dipartimento di Farmacia e Biotecnologie, Alma Mater Studiorum, Università di Bologna, Via Belmeloro 6, 40126 Bologna, Italy; matteo.micucci2@unibo.it (M.M.); roberta.budriesi@unibo.it (R.B.); roberto.gotti@unibo.it (R.G.); alberto.chiarini@unibo.it (A.C.); 3 Valsambro S.r.l., Via Cairoli 2, 40121 Bologna, Italy; carla.marzetti@valsambro.it

**Keywords:** *Acacia Catechu* Willd., colorectal cancer, apoptosis, HT-29 cells, ROS, mitochondrial membrane potential, catechins, polyphenols, natural compounds

## Abstract

The research for innovative treatments against colon adenocarcinomas is still a great challenge. *Acacia catechu* Willd. heartwood extract (AC) has health-promoting qualities, especially at the gastrointestinal level. This study characterized AC for its catechins content and investigated the apoptosis-enhancing effect in human colorectal adenocarcinoma HT-29 cells, along with its ability to spare healthy tissue. MTT assay was used to describe the time course, concentration dependence and reversibility of AC-mediated cytotoxicity. Cell cycle analysis and AV-PI and DAPI-staining were performed to evaluate apoptosis, together with ROS formation, mitochondrial membrane potential (MMP) changes and caspase activities. Rat ileum and colon rings were tested for their viability and functionality to explore AC effects on healthy tissue. Quantitative analysis highlighted that AC was rich in (±)-catechin (31.5 ± 0.82 mg/g) and (−)-epicatechin (12.5 ± 0.42 mg/g). AC irreversibly decreased cell viability in a concentration-dependent, but not time-dependent fashion. Cytotoxicity was accompanied by increases in apoptotic cells and ROS, a reduction in MMP and increases in caspase-9 and 3 activities. AC did not affect rat ileum and colon rings’ viability and functionality, suggesting a safe profile toward healthy tissue. The present findings outline the potential of AC for colon cancer treatment.

## 1. Introduction

Colorectal cancer (CRC) is one of the most common causes of tumour deaths worldwide [1]. In Europe, it is the second and the third most common form of cancer for women and men, respectively. Its occurrence and progression depend on multiple issues, among which family, age, gender and personal history constitute the major risk factors [2]. Standard treatments include surgery and chemotherapy. In the latter case, drugs induce DNA damage or initiate multiple signalling pathways, including cell cycle arrest, DNA repair, etc., leading to cancer cell death. The outcome of chemotherapeutic drugs in patients, however, is related to the cancer subtype, and often the effects of cytotoxicity, drug resistance and adverse reactions constitute overwhelming problems [3].

Natural products continue to provide leads for compounds endowed with pharmacological activities, especially those for treating many types of cancer [4]. A recent report highlighted that 49% of the small molecules approved in the area of cancer from the 1940s to 2014 were natural products or novel structures directly derived from them [5]. These compounds have cytotoxic properties owing to many different mechanisms of action, such as the inhibition of tumour cell growth accompanied by the induction of apoptosis, DNA damage, etc. Furthermore, anticancer drugs have greater potential to kill tumour cells if administered in combination with plant-derived compounds, and hopefully have less adverse effects. To explore this possibility, several clinical trials for various cancers were performed, including those for CRC [6].

*Acacia catechu* Willd. extracts have been used in traditional medicine for the treatment of several diseases. It possesses hepatoprotective, antipyretic, antidiarrheal, hypoglycaemic, anti-inflammatory, immunomodulatory, antinociceptive, antimicrobial, free radical scavenging and antioxidant activities [7,8,9,10]. Moreover, recent studies have demonstrated that *Acacia catechu* Willd. exerts spasmolytic and antispastic activities in vitro by interacting with calcium channels and muscarinic receptors, without affecting *Lactobacilli* and *Bifidobacteria,* the most represented intestinal species, suggesting that it may benefit patients suffering from diarrhoea [11]. *Acacia catechu* Willd. extract contains high amounts of flavonoids, such as flavan-3-ols, (+)-catechin, (−)-epicatechin, (−)-epicatechin-3-O-gallate and (−)-epigallocatechin-3-*O*-gallate [12]. These derivatives and related polyphenols possess apoptosis-inducing activity in several cancer cell lines [13]. Thus, this study investigates the effects of a preparation obtained by *Acacia catechu* Willd. heartwood by decoction (AC) on human colorectal adenocarcinoma HT-29 cell line in order to highlight its potential use in cancer therapy. As the capability of AC to spare the viability and functionality of normal tissue may be of clinical interest, this aspect was also investigated in rat ileum and colon rings. The results showed that AC has potential as an anti-cancer agent, as it exhibits irreversible anti-proliferative effects and induces intrinsic apoptosis, while sparing healthy tissue.

## 2. Results

### 2.1. AC Chemical Characterization

**Figure 1:**
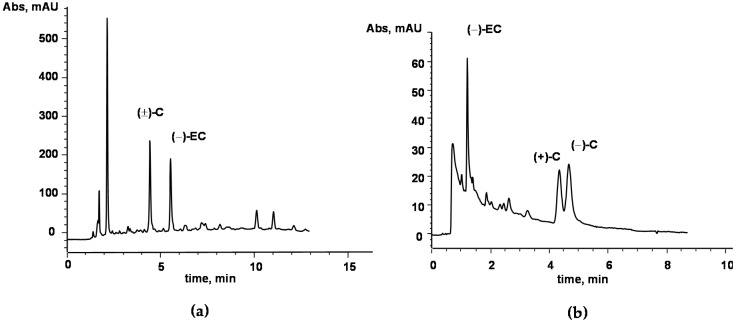
(**a**) HPLC chromatogram of a sample (aqueous extract) of *Acacia catechu* decoction. The method allows separation of the major catechins; namely, (±)-C, (−)-EC, (−)-ECG, (−)-EGC, (−)-EGCG and (−)-GCG. In the sample, only (±)-C and (−)-EC were found at levels higher than the limit of quantitation. (**b**) CD-MEKC of the same sample as in A). The method allows for the separation of the major catechins, as in the HPLC method. In addition, enantioseparation of (±)-C was achieved, revealing the presence of a significant amount of non-native (−)-C. HPLC and CD-MEKC conditions are described in the Materials and Methods section.

The decoction of *Acacia catechu* Willd. heartwood was characterized for its catechins content, assuming the most represented monomeric polyphenols as suitable phytomarkers for the herbal drug standardization [11,12]. The applied method, based on HPLC coupled with UV detection [14], showed the ability to resolve and quantify the major catechins in complex matrices; in Figure 1a the representative HPLC chromatogram of a sample of *Acacia catechu* decoction is reported. Identification of the catechins was performed by a comparison of the retention times of the analytical peaks in the sample with those of pure standards. Further confirmation of compounds’ identities was achieved by the standard addition method and by comparison of the online UV spectra acquired by the DAD detector. Among the considered monomeric catechins, only catechin ((±)-C) and epicatechin ((−)-EC), were found in the analyzed extract, whereas other major catechins were not detected at the sensitivity level of the method (limit of detection—LOD—within 1–10 μg/g, depending on the compound). By external standard quantitation based on peak area, (±)-C and (−)-EC were found to be 31.5 ± 0.82 mg/g and 12.5 ± 0.42 mg/g, respectively. Further characterization of the sample was carried out by means of a chiral method based on cyclodextrin-modified micellar electrokinetic chromatography (CD-MEKC) which had previously shown the ability to resolve catechin enantiomers [15]. Interestingly, among the major catechins occurring in plant kingdom, catechin is reported to be as the (+)-isomer with (2R, 3S) configuration and labelled as (+)-C. On the other hand, native epicatechin is the (−)-diastereomer (2R, 3R), labelled as (−)-EC. Since the *cis*-related compounds are thermodynamically less stable than the *trans*-related ones, epimerization of (2R, 3R) (−)-EC to the non-native (2S, 3R) (−)-C could easily occur in samples (e.g., processed food and herbal drugs), as was observed in the manufacturing of chocolate from Theobroma cacao [15]. The proposed CD-MEKC method, allowing the enantioresolution of (±)-C, was thus applied to the analysis of the aqueous extract of *Acacia catechu* Willd. decoction. Interestingly, the presence of both the enantiomers, namely, the native (+)-C and the artifact (−)-C, at approximately the same content level was observed (Figure 1b). The latter was assumed as a marker of the epimerization as the consequence of the process applied in decoction preparation (thermal treatment). Analysis of decoction preparations stored during very long-term period (at least two years) at room temperature in the dark, did not show loss of (−)-EC and (+)-C, nor epimerization progression, thus suggesting very high chemical stability of the preparation.

### 2.2. AC Induced a Concentration-Dependent, but not Time-Dependent, Irreversible HT-29 Cell Death

**Figure 2:**
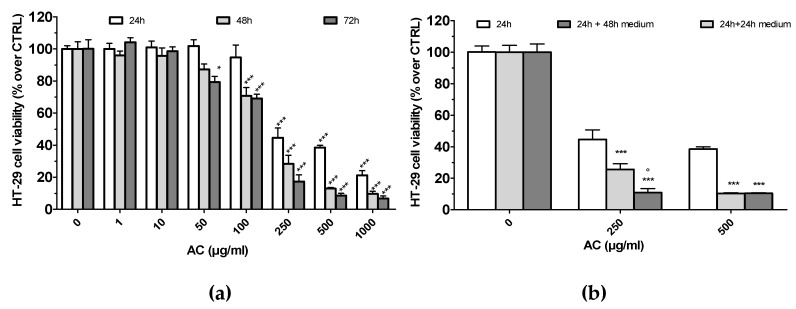
(**a**). Human colorectal adenocarcinoma HT-29 cells’ viability after treatment with *Acacia catechu* Willd. extract (AC) for 24, 48 and 72 h. Panel B. Reversible or irreversible cytotoxic effect caused by AC. HT-29 cells were treated with AC (250 and 500 µg/mL, 24 h). Afterward, AC was washed off and cells were incubated with fresh serum-containing medium for an additional 24 or 48 h. After each time point, MTT test was performed as detailed in Materials and Methods. In both panels, values are means ± SEMs of 4 or 5 independent experiments in which four points/concentrations/times were run; controls (AC 0 µg/mL) represent untreated cells. Statistical significance was assessed by ANOVA followed by Dunnett post-test. (**a**): * *p* < 0.05, *** *p* < 0.001 vs. controls, same time point. (**b**): *** *p* < 0.001 vs. 24 h AC-treated cells; ° *p* < 0.05 vs. 24 h + 24 h medium-treated cells.

Cytotoxic effects towards human colorectal adenocarcinoma HT-29 cells were assessed after 24, 48 and 72 h of AC treatment (0.01–1000 µg/mL) by using an MTT assay. The results showed that HT-29 cell viability was not affected up to 10 µg/mL, whereas it decreased significantly with AC 50 µg/mL (for 72 h), 100 µg/mL (48 h) and 250 µg/mL up to 1000 µg/mL (24 h) (Figure 2a). This effect was concentration-dependent, but not time-dependent, as IC_50_ values among the selected time points were almost comparable (IC_50_ 24 h 199.4 ± 10.3 µg/mL; 48 h, 156.1 ± 11.3 µg/mL; 72 h, 139.5 ± 11.2 µg/mL).

Changes in HT-29 cell morphology mirrored MTT results. At variance with HT-29 untreated cells, which grow normally as tight colonies, those treated with AC (250 and 500 µg/mL, 24 h) showed significant changes in both number and size; displayed larger extracellular spaces; and began to shrink, round and fragment, thus resembling an apoptotic cell’s typical appearance. These changes were progressively evident upon the increase in AC concentration, and cytotoxicity grades 2, 3 (250–500 µg/mL) and severe—grade 4 (1000 µg/mL)—occurred (see Figure S1).

To check whether the AC growth-inhibitory effect was reversible, cells were initially exposed to the extract for a 24 h period, following which they were washed thoroughly to remove the treatment, and were cultured in complete drug-free medium for a further period; i.e., 24 or 48 h. AC concentrations of 250 and 500 µg/mL, which caused grade 2 or 3, of toxicity were tested. As reported in (b) of Figure 2, AC caused an irreversible cytotoxic effect at both 250 and 500 µg/mL, as a drastic drop in the viability was evident even after an extra 48 h period of incubation with drug-free medium.

### 2.3. AC Induced an Apoptosis-Mediated HT-29 Cell Death

**Figure 3:**
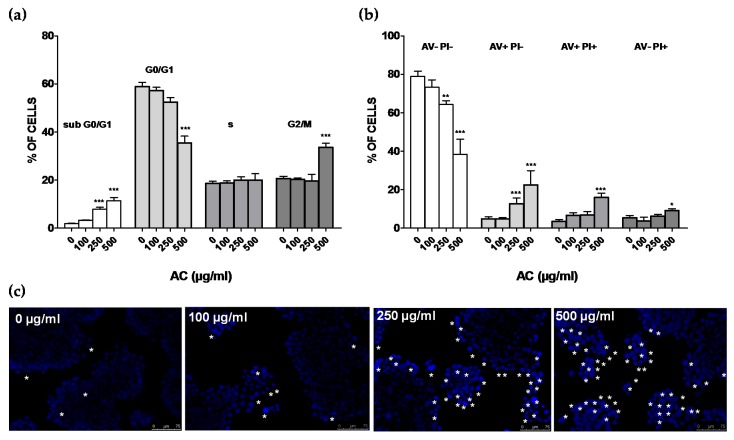
(**a**). *Acacia catechu* Willd. extract (AC)-mediated effects on the human colorectal adenocarcinoma HT-29 cell cycle. Percentages of cells in subG0/G1, G0/G1, s and G2/M phases. (**b**). Apoptotic cells’ detection by double staining with annexin V (AV) and propidium iodide (PI). Values are means ± SEMs of four or five independent experiments in which four points/concentrations were run; controls (AC 0 µg/mL) represent untreated cells. * *p* < 0.05, ** *p* < 0.01, *** *p* < 0.001 vs. controls (ANOVA followed by Dunnett post-test). (**c**). DNA condensation and damage assessed by DAPI staining. Asterisks indicate cells with fragmented nuclei and condensed DNA, considered apoptotic. Each photograph was representative of three independent observations (scale bar 75 μm).

To study in more detail the mechanisms causing cell death observed after 24 h of treatment with AC, flow cytometry-mediated cells cycle analysis was performed. AC concentrations of 250 and 500 µg/mL (grade 2 or 3 of toxicity), along with 100 µg/mL—taken as the highest AC safe concentration, were used. A concentration-dependent rise in sub-G0/G1 hypodiploid cells, accompanied by a reduction in those in the G0/G1, was observed upon AC treatment (Figure 3a). While 100 µg/mL did not affect cell cycle distribution, 250 µg/mL increased apoptotic cells (subG0/G1 +9.9%, *p* < 0.01 vs. control), reducing, at the same time, although not significantly, those in G0/G1(−6.4%, *p* > 0.05 vs. control). Accordingly, 500 µg/mL AC had the most striking effect, as subG0/G1 (+11.5%, *p* < 0.01 vs. control), G0/G1 (−23.4%, *p* < 0.01 vs. control) and G2/m (+13.0%, *p* < 0.01 vs. controls) cells were affected. Apoptosis was further investigated with the AV-PI assay (Figure 3b). Challenge with AC caused a marked increase in early apoptotic (+7.8 and +17.8%, *p* < 0.01 vs. control for 250 and 500 µg/mL AC, respectively), late apoptotic (+12.6 %, *p* < 0.01 vs. control for 500 µg/mL AC) and necrotic HT-29 cells (+5.0%, *p* < 0.05 vs. control for 500 µg/mL AC). This effect was accompanied by progressive reduction in AV- and PI-negative cells, scored as healthy. DAPI staining supported characteristic apoptotic changes elicited by AC, such as chromatin condensation, nuclear pyknosis, elevated number of nuclear body fragments and irregular edges around the nucleus, which increased in a concentration-dependent manner (indicated by asterisk in Figure 3c).

### 2.4. AC Caused ROS Formation along with Loss in Mitochondria Membrane Potential

**Figure 4:**
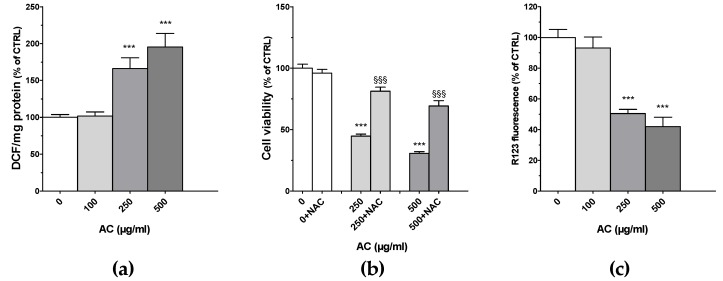
*Acacia catechu* Willd. extract (AC)-mediated effects on 2′,7′-dichlorofluorescein’s (DCF) intracellular concentration (**a**); cell viability in the presence of N-acetyl-L-cysteine (NAC) (**b**) and rhodamine 123 (R123) staining (**c**) in human colorectal adenocarcinoma HT-29 cells. NAC (1 mM) was pre-incubated for 2h before the addition of AC (250 or 500 µg/mL). Data are reported as means ± SEMs of at least four independent experiments in which four points/concentrations were run; controls (AC 0 µg/mL) represent untreated cells. *** *p* < 0.001 vs. controls; ^§§§^ *p* < 0.01 vs. the same concentration of AC (ANOVA followed by Dunnett post-test).

Many chemotherapy drugs cause cell apoptosis by inducing the formation of ROS, which in turn further stimulate cell apoptosis and DNA damage [16]. For this reason, ROS formation was assessed by monitoring the conversion of the non-fluorescent 2′,7′-dichlorofluorescin to fluorescent 2′,7′-dichlorofluorescein (DCF). Results showed that upon AC treatment, a huge increase in DCF occurred, suggesting a considerable formation of ROS (Figure 4a). This effect, however, was almost comparable between 250 and 500 µg/mL AC concentrations (+166.2 ± 14.3 and +195.5 ± 18.3, respectively). To further support the role of ROS in the cytotoxic effects of AC, the ROS scavenger *N*-acetyl-L-cysteine (NAC, 1 mM) was used. As reported in Figure 4b, HT-29 cells’ viability was significantly recovered in the presence of NAC, suggesting that ROS play a key role in AC-mediated HT-29 cell death. As one of the early events in apoptosis consists of the alteration of mitochondrial membrane integrity, changes in the mitochondria membrane potential (MMP) of AC-treated cells were assessed by staining them with R123. This dye selectively enters mitochondria with an intact membrane potential and there is retained, unless MMP is lost, causing R123 to be washed out from the cells. As reported in Figure 4c, intracellular R123 fluorescence decreased significantly by about 50–60% for both 250 and 500 µg/mL AC concentrations.

### 2.5. AC-Induced Changes in Caspase Activity

Different pathways of apoptosis are involved in the induction of cell death, including the mitochondria-mediated and the extrinsic receptor-mediated pathways, within which caspase-9 and 8 play essential roles, respectively. These, in turn, activate caspase-3 and fragmentation of DNA [17,18]. To investigate the involvement of these caspases, AC-treated HT-29 cell lysates were used to perform fluorescence assays with specific caspase-3, 8 and 9 substrates.

**Figure 5:**
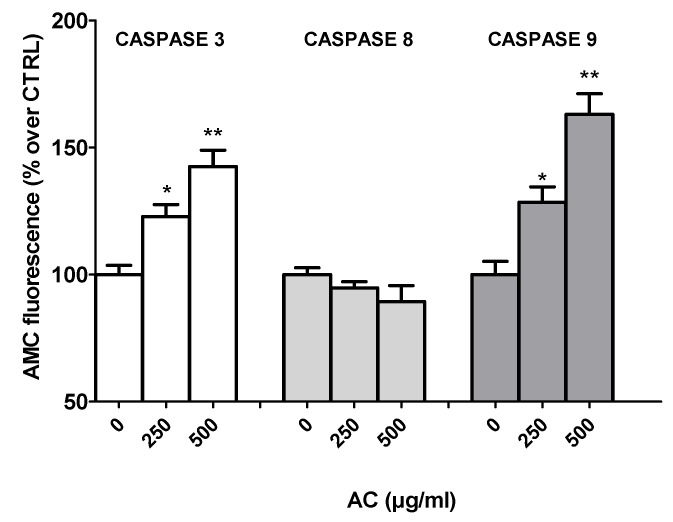
*Acacia catechu* Willd. extract (AC)-mediated effects on caspase-3, 8 and 9 activities in human colorectal adenocarcinoma HT-29 cells. The specific fluorogenic substrates DEVD-AMC (caspase-3), IETD-AMC (caspase-8) and LEHD-AMC (caspase-9) were used, and so was the fluorescence of the AMC-fragment released by active caspases measured at 380 and 460 nm excitation and emission wavelengths, respectively. Data are reported as means ± SEMs of at least three independent experiments in which four points/concentrations were run, and controls (AC 0 µg/mL) represent untreated cells. * *p* < 0.05, ** *p* < 0.01 vs. controls (ANOVA followed by Dunnett post-test).

Caspase-3 plays a central role in apoptotic responses. As shown in Figure 5, the activities of both cleaved caspase-3 and 9 were significantly increased in AC-treated HT-29 cells, in contrast with that of caspase-8, which was not affected by the treatment, suggesting the activation of the mitochondrial pathway, rather than extrinsic receptor-mediated apoptosis pathway.

### 2.6. AC Does not Affect Viability and Functionality of Healthy Rat Ilia and Proximal Colon Rings

For drugs targeting cancer cells, it is crucial to avoid toxicity to healthy tissues. For this reason, the effects of AC on rat proximal colon tissue viability was assessed.

Ilia were tested as well, as small intestine tissue constitutes the largest part of the gastro-intestinal tract. As in rat ileum and colon rings, a lower amount of AC might be attained in the extracellular milieu with respect to a cell monolayer, AC concentration was raised up to 1000 µg/mL.

**Figure 6:**
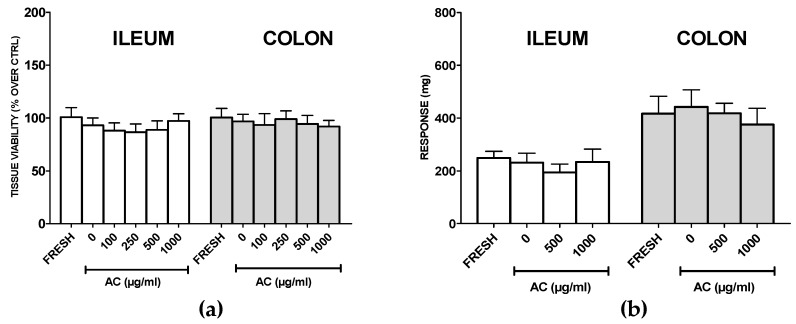
Rat ileum and proximal colon rings’ viabilities (**a**), and high potassium-evoked contraction (KCl 60 mM) (**b**) after treatment with *Acacia catechu* Willd. extract (AC) for 24 h. Data are reported as means ± SEMs of at least three independent experiments in which four rings/concentrations were run; FRESH and AC 0 µg/mL refer to rings immediately after being explanted or treated with PSS for 24 h, respectively.

Results showed that 24 h of treatment with AC did not change the viabilities of the ilia or proximal colon rings of rats (Figure 6a). Interestingly, the highest concentration of AC did not affect tissue contractility evoked by high potassium-containing solution (Figure 6b), thus suggesting a safe profile of AC toward healthy tissue.

## 3. Discussion

Natural products constitute an important source of new anticancer molecules, thereby driving a novel direction for the prevention and therapy of cancers [6]. Natural product-derived drugs exert anticancer effects by hampering metastasis and angiogenesis and promoting apoptosis, which are the most important features of human cancers [6,18]. Moreover, drugs derived from natural products are generally endowed with better bioactivities and lower toxicity, and some of them have been combined with conventional therapies to enhance cancer cells’ susceptibility [19].

This study investigates the apoptosis-enhancing effect of AC on the human colorectal adenocarcinoma HT-29 cell line in order to highlight its potential use in CRC therapy. As a first step, the decoction of *Acacia catechu* Willd. heartwood was characterized for its catechins content, and only catechin (±)-C and epicatechin (−)-EC were found to be present. It is noteworthy that the amounts of these two catechins (total ~40 mg/g) are comparable and even higher than those reported in green tea [20], whose mean contents are in the order of 6 mg/g and 1.5 mg/g for (−)-EC and (±)-C, respectively, as determined in about one hundred analyzed samples [21]. This, together with the observation that the bark extract’s catechin content is less subjected to seasonal variation with respect to that in green tea or some fruits [22,23], makes AC a very interesting source of active polyphenols.

Biological assays showed that AC affected HT-29 cell viability in a concentration, but not time dependent fashion. When AC treatment time increased from 24 to 48 and 72 h, in fact, IC_50_ values remained in the range of 190–140 µg/mL AC concentration (i.e., 26–19 µM catechin content). This effect can be explained by considering that treated HT-29 cells undergoing apoptosis after 24 h of treatment (see below) may have progressed into necrosis due to the prolonged incubation with AC. Interestingly, comparable) values for MTT assay, cell cycle analysis and DAPI staining were found in Caco2 cells (see Figure S2), suggesting that this other widely-used colorectal adenocarcinoma cell line, characterized by high homology to enterocytes in the intestinal epithelium, is equally affected by AC.

In the case of cancer cells, is mandatory to investigate whether a cell line may (or may not) be able to restart its proliferating activity upon drug treatment. It is in fact crucial to demonstrate the presence of the so-called “point of no return,” a limit line between cell injury and cell death; overtaking that causes irreversible damage. To assess whether AC-mediated cytotoxicity was reversible or irreversible, HT-29 cells were treated for 24 h with the extract, which was then washed off, and incubated with fresh serum-containing medium for an additional 24 or 48 h. The results suggested that AC elicited an irreversible cytotoxic effect against HT-29 cells, since a drastic decrease in the viability occurred even after incubation with drug-free medium for an extra 48 h period. Moreover, flow cytometric analysis and DAPI staining indicated that cytotoxic effects mostly consisted of apoptosis-mediated cell death. Early and late apoptotic, and some necrotic cells were in fact highlighted by AV-PI assay upon AC treatment—confirmed also by cytoplasmic shrinkage, membrane blebbing observed by contrast phase microscopy, DNA fragmentation (DAPI assay) and an increase in sub G0/G1 cells (cell cycle analysis).

Following the induction of apoptosis, perturbation of mitochondrial membrane potential is one of the main and earliest intracellular events. Mitochondria are the key regulators of the mechanisms which control the cell’s survival/death balance, since they are the main source of cellular ROS and ATP [6,18]. Consequently, the production of ROS in treated cells was assessed, and results showed that a significant rise in their formation occurred upon AC challenge.

Excessive ROS production is a key negative element that results in the failure of suppression of antiapoptotic factors, thereby further triggering apoptosis. The fluorescent probe R123 was thus used to investigate the effects of elevated ROS production on the function of MMP in treated HT-29 cells. A drop in MMP, leading to the membrane depolarization of the mitochondria, was demonstrated, suggesting that the induction of apoptosis by AC may be associated with the activation of the mitochondrially-mediated pathway. The effects of AC on ROS and MMP, however, were comparable among 250 and 500 µg/mL, indicating that a plateau was reached. This plateau effect was also reported for human colon cancer HT-29 cells exposed to 30–100 μM of EC [24], a concentration comparable to those used in the present study—likewise for human colon carcinoma LoVo cells exposed to very high (500–1000 μM) levels of ECG [25,26].

ROS formation can be explained by considering that AC catechins can auto-oxidize to generate ROS in cell culture medium, and in turn, cause cell death [27,28]. Polyphenols, in fact, have the potential to promote the autoxidation of phenolic hydroxyl groups [29], and that mechanism is at the basis of their antibacterial and anti-cancer activities [28]. In particular, catechins can selectively kill cancer cells by promoting ROS generation over a critical threshold and by mobilization of endogenous chromatin-bound copper ions [30]. Among catechins, EGCG from green tea is one of the most studied, and it has been reported to be active against many types of cancer, including colorectal [31], lung [32], breast [33] and liver [34]. These effects basically arise from its abilities to promote pro-apoptotic effects, inhibit angiogenesis, regulate cellular metabolic pathways, and reduce inflammatory factors [28,35]. EGCG can increase the activities of traditional anticancer treatments [36,37,38] and reverse drug cell resistance, and this has encouraged its use in clinical trials for treatments of various types of cancer and other diseases [39]. *Acacia catechu* Willd. contains a variety of catechin monomers differently distributed in heartwood, leaves and chunks resin, (−)-EC and (+)-C being reported as the most abundant [12]. Accordingly, the heartwood extract used in the present study showed the presence of (−)-EC and (±)-C, as outlined by the quantitative analysis performed. Their pharmacological properties, although much less investigated with respect to EGCG, include the capability of inducing apoptosis [40], an effect strictly linked to their prooxidant activity [41,42,43]. Catechins, as previously outlined, can behave as pro-oxidants in the presence of Cu(II), leading to cytotoxic action [41]. As the cellular copper level is considerably elevated in cancer cells [43,44], it is conceivable that in the present experimental conditions, HT-29 cells may be more subjected to electron transfer between copper ions and AC catechins to generate ROS. Since cancer cells, which have altered antioxidant systems, are under constant oxidative stress because of increased rate of growth and metabolism [45,46], further ROS formation generated by AC catechins can overwhelm the HT-29 cells’ antioxidant capacity, leading to irreversible damage and apoptosis. This was supported by the fact that the decrease in cell viability was significantly reverted by the ROS scavenger NAC, thereby suggesting that ROS formation may be necessary in AC-mediated effects. The copper-dependent ROS formation could also explain the previously discussed plateau effect, as a further increase in ROS might not occur upon increased AC concentration due to limited copper availability. The possibility that catechins may also induce the production of ROS by different mechanism(s), which could involve the electron transport chain in the mitochondria [47], or catechin-mediated hydrogen-peroxide formation [48], however, cannot be ruled out.

The activation of caspases, a family of cysteine-aspartic-acid specific proteases, is a critical event in the induction of apoptosis, and this ultimately leads to the hallmarks of apoptosis itself, such as chromatin condensation, DNA fragmentation and plasma membrane blebbing [17,18].

The mitochondria-dependent pathway of cell death involves the activation of the downstream caspase-9 via apoptosome formation, which leads to active caspase-3 and 7, the most effective caspases with many cellular targets [17,18]. This hypothesis was confirmed by the fluorimetric caspase assays, which showed increases in caspase-9 and caspase 3 activities following AC treatment.

Apoptosis can occur by the so-called extrinsic pathway as well, mediated by death receptors. This implies the activation of caspase-8, which cleaves and activates the above-mentioned downstream executioner caspases [17,18]. In the present study, however, AC treatment did not affect caspase-8’s activity, thereby confirming that the apoptosis induced in HT-29 cells was mediated via the intrinsic mitochondrial pathway and not via the extrinsic, death receptor-linked caspase-8 pathway. Finally, even though intrinsic apoptotic signalling is potentially triggered by oxidative stress in many catechin-treated cancer cells [40,41,42,43], the possibility that AC activates apoptosis via a ROS-independent mechanism should be studied further.

Taken all together, the evidence from MTT assays, AV-PI and DAPI staining, cell cycle analysis, ROS production, MMP changes and the activation of caspases 3 and 9, demonstrated AC’s promising pro-apoptotic activity towards the human colorectal adenocarcinoma HT-29 cell line via the intrinsic mitochondrial pathway, suggesting that it might be useful as a support to CRC treatment or prevention. Orally administered drugs are exposed to a changing environment, and even if they are planned to target the colon, they will be in direct contact with both the upper part of the GI tract and the colon itself. It is therefore mandatory that healthy tissue is not affected by the treatment. The effects of AC on rat ileum and proximal colon viability and functionality were thus assessed, and results highlighted the safe profile of AC, indicating a selective activity against cancer tissues. This sparing activity was already reported for EGCG and EGC in many cancer cells lines [49,50,51,52,53], and although the precise mechanism is still debated, the lower intracellular copper content of non-malignant cells might constitute the key difference [54]. Finally, AC is endowed with antibacterial activity against Gram-negative (*Escherichia coli, klebsiella pneumoniae, Proteus mirabilis and Pseudomonas aeruginosa*) and Gram-positive bacteria (*Staphylococcus aureus and Streptococcus pneumonia*) [11,55]; at the same time it is ineffective toward *Lactobacilli* and *Bifidobacteria*, the most represented intestinal species. The human gastrointestinal microbiota has a key role in human health, as dysbiosis is associated with various disorders and many types of cancer, including CRC [56]. Thus, the ability of AC to spare microbiota, together with the general safety profile of catechins and epicatechins, increases the therapeutic potential of AC. Moreover, it was recently reported that plasma concentrations of catechin and epicatechin quickly peaked after being orally administered AC in rat, with a rapid and wide distribution in all tissues, especially the intestine [57], reaching amounts comparable to those revealed to be effective in the present study. Thus, by using the translational dose calculation of Reagan–Shaw [58], an extrapolated dose for a man of about 10 mg/kg of catechin and epicatechin is attained, an amount which makes the administration of AC extract feasible for possible future clinical implications.

## 4. Materials and Methods

### 4.1. Plant Materials

A preparation obtained from *Acacia catechu* Willd. heartwood by decoction (AC) was supplied by BIO-LOGICA S.R.L. (via della Zecca 1, 40100 Bologna, Italy). The hydroalcoholic fluid extract production from the plant *Acacia catechu* Willd. was obtained by maceration and percolation according to European Pharmacopoeia 8.0. For more insight, please visit Minardi (A. Minardi and figli s.r.l. via Boncellino 18/A 48012 Bagnacavallo (RA) Italy; website, www.minardierbe.it).

### 4.2. Phytochemical Analysis

#### 4.2.1. Chemicals

Catechins standard references, (±)-catechin hydrate ((±)-C), (+)-catechin ((+)-C), (−)-epicatechin (−)-EC, (−)-epigallocatechin (−)-EGC, (−)-epicatechin gallate (−)-ECG, (−)-epigallocatechin gallate (−)-EGCG, (−)-gallocatechin gallate (−)-GCG, sodium dodecyl sulphate (SDS) and (2-hydroxypropyl)-β-cyclodextrin (HP-βCD, degree of substitution ~0.6). Boric acid, phosphoric acid, sodium hydroxide, trifluoroacetic acid (TFA), acetonitrile (HPLC grade) and all the other chemicals, were purchased from Sigma-Aldrich (Milan, Italy, www.sigmaaldrich.com). Water used for preparation of standard solutions, running buffers and HPLC mobile phases, was purified by a Milli-Q apparatus (Millipore, Milford, MA, USA, www.merckmillipore.com).

#### 4.2.2. HPLC Method

Aliquots of 100 mg of AC decoction were extracted with 10 mL water in an ultrasonic bath at room temperature for 15 min. The filtered solution was diluted 1/1 (v/v), with water and analyzed by HPLC under reversed-phase conditions. A Liquid Chromatograph by Agilent 1050 Ti series (Agilent Technologies, Waldbronn, Germany, www.agilent.com) equipped with a DAD detector (detection was set at 280 nm) was used. The stationary phase was a core-shell type Kinetex PFP (pentafluoro-phenyl) 150 × 4.6 mm (5 μm, 100 Å) by Phenomenex (Castelmaggiore, Bologna, Italy, www.phenomenex.com). The mobile phase was composed of acetonitrile (A) and aqueous TFA 0.1% (v/v) (B) under gradient elution: from 10/90 (A/B) to 20/80 (A/B) in 10 min at the flow rate of 1 mL/min. Sample injections were manually done by a Rehodyne Model 7125 injector (volume 20 μL).

Calibration graphs and sensitivity. According to a previous method [14] the calibration was carried out for the catechins: (−)-ECG (2.5–250 µg/mL), (−)-EGCG (25–500 μg/mL), (−)-EC (5–200 μg/mL), (−)-EGC (10–300 μg/mL), (±)-C (0.3–50.0 µg/mL) and (−)-GCG (2–50 μg/mL) in the concentration ranges given in brackets. Triplicate injections were made for each calibration point and the peak areas of the analytes were plotted against the concentrations of each of corresponding compounds; the determination coefficients were found to be higher than 0.9990. The sensitivity data as limits of detection (LODs) and limits of quantitation (LOQs) were determined by diluting standard solutions till signal-to-noise ratios of 3:1 and 10:1, respectively. The values were in the ranges 0.01–0.1 µg/mL (LOD) and 0.05–0.3 µg/mL (LOQ), depending on the compound.

#### 4.2.3. Capillary Electrophoresis Method (Cyclodextrin-Modified Micellar Electrokinetic Chromatography, CD-MEKC)

Electrophoretic experiments were performed by a HP^3D^CE instrument by Agilent Technologies. Fused-silica capillaries (50 µm id, 30 cm total length, 8.5 cm length to the detector) were from CM Scientific Ltd. (Ryefield Way Silsden, UK, www.cmscientific.com). The separations were performed at a constant voltage of 15 kV, and the cartridge temperature was 25 °C. The detection was carried out by using the on-line DAD detector, and the quantitation was performed at the wavelength of 200 nm. Hydrodynamic injections were performed at 25 mbar for 5 s. New capillaries were conditioned by flushing sequentially 1M sodium hydroxide, 0.1 M sodium hydroxide and water in the order, for 10 min each. Between the injections the capillary was rinsed with 0.1M sodium hydroxide, water and running buffer for 3 min each.

Borate-phosphate buffer was used as the background electrolyte (BGE); it was prepared at a concentration of 12.5 mM and pH 2.5 by following a standard procedure. The obtained buffer was then supplemented with SDS (90 mM) and HP-βCD (25 mM).

Calibration graphs and sensitivity. According to a previous method [11,15], the calibration was carried out for the catechins: (−)-EC (5.0–300.0 µg/mL), (+)-C and (−)-C (0.3–50.0 µg/mL) in the concentration ranges given in brackets. Triplicate injections were made for each calibration point, and the peak areas of the analytes were plotted against the concentrations; the determination coefficients were found to be higher than 0.9990. The sensitivity data LOD and LOQ were determined by diluting standard solutions till signal-to-noise ratios of 3:1 and 10:1, respectively. The values were for (−)-EC, (−)-C and (+)-C: 0.1 µg/mL (LOD) and 0.4 µg/mL (LOQ).

### 4.3. Cell Cultures, AC Treatments and Cell Viability Assay

Human colorectal adenocarcinoma HT-29 cells (ATCC^®^ HTB-38™, passages 10–20) were grown in a humidified atmosphere of 95% air and 5% CO_2_ at 37 °C as previously reported [59]. AC was prepared immediately before use as 10 mg/mL stock solution in PBS, and pH adjusted to 7.5 before dilution to the desired final concentration. The solution was sterile filtered by passage through a 0.2-micron sterile filter. To assess AC effects, HT-29 cells (5 × 10^3^ cells/well, final volume 200 µL) were treated with the extract (0–500µg/mL) for 24, 48 or 72 h, renewing AC solution every 24 h. To assess the role of ROS in AC-mediated cytotoxicity, N-acetyl-L-cysteine (NAC, 1 mM) was pre-incubated for 2h, and then AC (250 or 500 µg/mL) was added and left for 24 h. At the end of the treatments, MTT (20 µL of 5 mg/mL solution in PBS) was added to each well, and the assay was performed as already described [60]. Reversibility of AC cytotoxicity was tested by treating cells for 24 h with the extract, adding then fresh AC-free culture medium and assessing cell viability after a further 24 or 48 h of incubation [61].

### 4.4. Cells Morphological Assays

Apoptotic cells experiencing damage in the nuclei are featured by cell shrinkage, membrane blebbing and the presence of apoptotic bodies. In order to monitor these changes caused by AC, HT-29 cells were examined by using a phase-contrast light microscope, and the results were evaluated using the grade scale described in USP 28 (United States Pharmacopeia edition 2005) (grades 0–4) for the assessment of the cytotoxic potentials of the materials as follows: grade 0—no reactivity (discrete intracytoplasmic granules, no cell lysis); grade 1—slight reactivity (no more than 20% of the cells are round, loosely attached and without intracytoplasmic granules; occasional lysed cells are present); grade 2—mild reactivity (no more than 50% of the cells are round and devoid of intracytoplasmic granules; no extensive cell lysis and empty areas between cells); grade 3—moderate (up to 70% of cells are rounded or lysed); grade 4—severe (nearly complete destruction of the cells). [62,63].

### 4.5. Apoptosis Assays

Flow cytometry techniques, such as cell cycle and sub-G0/G1 population-analysis, and annexin V/propidium iodide (AV/PI) and 4′,6-diamidino-2-phenylindole (DAPI) staining, were applied to identify apoptotic cells. HT-29 cells (5 × 10^5^ cells/well, final volume of 2 mL) were treated with AC for 24 h, and then the analysis was performed by using protocols previously described [62]. Alexa fluor 488™-AV/PI double staining kit (Life Tecnologies Italia, Monza, Italy) was used to detect the externalization of phosphatidylserine in apoptotic cells [64]. Samples were analysed on a FACScan flow cytometer (BD Biosciences, San Jose, CA, USA) by using CellQuest software v. 3.0 (BD Biosciences, San Jose, CA, USA). Viable cells were both AV and PI-negative; cells in early apoptosis were AV-positive and PI-negative; cells in late apoptosis were both AV and PI-positive; necrotic cells were PI-positive and AV-negative. Apoptosis was assessed also by analysing changes in nuclear morphology with the DAPI staining kit (Life Tecnologies Italia, Monza, Italy), as previously described [62].

### 4.6. ROS Detection

HT-29 cells were seeded into 6-well plates at 5 × 10^5^ cells/well, grown for 24 h under standard conditions and then treated with AC for 24 h. ROS generation was assessed in cells rinsed with PBS and loaded with 10 μM 2′,7′-dichlorofluorescin diacetate (DCFDA) for 10 min at 37 °C, then washed, centrifuged at 13,000× *g* for 5 min and re-suspended in 0.7 mL of PBS. The intracellular fluorescence (504 nm excitation, 529 nm emission, Fluoroskan Ascent fluorimeter, Thermo Labsystems, Helsinki, Finland), was normalized to mg of cellular protein of the samples and expressed as percent of untreated-, control-cells [65].

### 4.7. Rhodamine-123 Staining

The fluorescent probe rhodamine-123 (R123) was used to check for mitochondria integrity [64]. This dye selectively enters mitochondria with an intact membrane potential and here is retained, unless MMP is lost, thus causing R123 washed out of from the cells. After AC treatment, HT-29 cells were stained with R123 (4 µM) for 10 min at 37 °C in the dark and then thoroughly washed with PBS. Afterward the fluorescence of 10000 single cells/sample was measured by FACScan flow cytometer (BD Biosciences, San Jose, CA, USA) at 505 nm (R123). CellQuest software v. 3.0 (BD Biosciences, San Jose, CA, USA) was used for intracellular fluorescence determination.

### 4.8. Caspase Activity

At the ends of the treatments, cells were added with 500 µL of caspase lysis buffer (20 mmol/L Hepes/KOH, 10 mmol/L KCl, 1.5 mmol/L MgCl_2_, 1 mmol/L EGTA, 1 mmol/L EDTA 1, 1 mmol/L DTT, 1 mmol/L PMSF and 10 μg/mL leupeptin, pH 7.5). Afterward, cell lysates (20 µg proteins) were incubated for 1 h at 37 °C with of the following fluorogenic substrates (Enzo Life Sciences, Farmingdale, NY): caspase-9 LEHD-AMC (Ac-Leu-Glu-His-Asp-7-amino-4-methylcoumarin; AMC, 7-Amino-4-methylcoumarin); caspase-3 DEVD-AMC (Ac-Asp-Glu-Val-Asp-7-amino-4-methylcoumarin); caspase-8 IETD-AMC (Ac-Ile-Glu-Thr-Asp-AMC). These were used at 20 μM in 0.25 mL of caspase assay buffer (25 mmol/L Hepes, 0.1% *w/v* CHAPS, 10% *w/v* sucrose, 10 mmol/L DTT, 0.01% *w/v* egg albumin, pH 7.5). The reaction was stopped by adding 0.1% *w/v* ice-cold trichloroacetic acid (0.75 mL) and the fluorescence of AMC fragment released by active caspases was then read (Fluoroskan Ascent fluorimeter, ThermoLabsystems, Helsinki, Finland) at 380 nm and 460 nm (excitation and emission wavelengths, respectively) [66].

### 4.9. Rat Ileum or Colon Rings

All animal care and experimental protocols conformed to the European Union Guidelines for the Care and the Use of Laboratory Animals (European Union Directive 2010/63/EU, http://ec.europa.eu/environment/chemicals/lab_animals/home_en.htm) and were approved by the Italian Department of Health (666/2015-PR). Male Wistar rats (250–350 g; Charles River Italia, Calco, Italy) were used: 0.5 cm ileum and proximal colon rings were prepared according to the protocols of [67,68]. In particular, rings were placed in a water-jacketed (37 °C) organ bath containing 10 mL of modified Krebs–Henseleit physiological salt solution (PSS) (composition in mM: 118 NaCl, 4.75 KCl, 2.5 CaCl_2_, 1.19 MgSO_4_, 1.19 KH_2_PO_4_, 25 NaHCO_3_, and 5.5 glucose, bubbled with a 95% O_2_–5% CO_2_ gas mixture, pH 7.4) and mounted on two stainless steel wires, one of which was connected to a force transducer that measured isometric tension in order to evaluate their contractility. Ileum or proximal colon rings were then equilibrated for 90 min under a resting tension of 1.0 g and were contracted by PSS containing high potassium (KCl 60 mM). Contractile isometric tension was recorded and analysed by means of a PowerLab data acquisition system and LabChart 7.3.7 Pro (Power Lab; ADInstruments, Castle Hill, Australia) for rings prepared immediately after their explant (“fresh” controls), and those treated for 24 h with PSS (controls, 0 µg/mL AC) or AC 500-1000 µg/mL [67].

Viability of tissue was also assessed by using MTT assay [69]. Rings were placed in 24 multiwell, one ring/well, and incubated for one hour at 37 °C with 800 µl of MTT solution (0.5 mg/mL). Afterward rings were transferred to a 96 MW (one ring/well) and 800 μL of DMSO added. The plate was stirred for 30 min to allow for the solubilization of the formazan salts formed. Subsequently, 100 μL of supernatant was taken and the absorbance assessed (560–630 nm) by using a plate reader (Multiskan TM GO, Thermo Scientific, Waltham, MA, USA).

### 4.10. Analysis of Data

Data were collected as quadruplicates from at least four independent experiments. The results were expressed as means ± SEMs. Cell viability was expressed as percentage of untreated cells (controls). Statistical significance was assessed by using ANOVA followed by Dunnett *post test* (GraphPad Prism version 5.04, GraphPad Software Inc., San Diego, CA, USA). In all comparisons, the level of statistical significance (*p*) was set at 0.05.

## 5. Conclusions

The present study outlines the potential of AC for CRC treatment, as this extract induced cytotoxicity of human colorectal adenocarcinoma HT-29 cells, which was accompanied by increases in apoptotic cells and ROS formation; a reduction in MMP; and increases in caspase-9 and 3 activities. AC did not affect rat ileum and colon rings viability and functionality, suggesting a safe profile toward healthy tissue. Moreover, AC main components are absorbed rapidly and eliminated slowly [57], and this might constitute an added value to the potential use of AC for CRC prevention.

## Supplementary Materials

Supplementary materials can be found at https://www.mdpi.com/1422-0067/21/6/2102/s1.

## Abbreviations

AC	*Acacia catechu* Willd. heartwood extract
AMC	7-amino-4-methylcoumarin
AV	annexin V
C	catechin
CRC	colorectal cancer
DAPI	4′,6-diamidino-2-phenylindole
DCFDA	2′,7′-dichlorofluorescin diacetate
DEVD-AMC	Ac-Asp-Glu-Val-Asp-7-amino-4-methylcoumarin
EC	epicatechin
ECG	epicatechin gallate
EGC	epigallocatechin
EGCG	epigallocatechin gallate
IETD-AMC	Ac-Ile-Glu-Thr-Asp-7-amino-4-methylcoumarin
LEHD-AMC	Ac-Leu-Glu-His-Asp-7-amino-4-methylcoumarin
MMP	mitochondrial membrane potential
MTT	4,5-dimethylthiazol-2-yl)-2,5-dipheniltetrazolium bromide
PI	propidium iodide
R123	rhodamine-123
