# Supplementary material for: Apoptotic-Induced Effects of Acacia Catechu Willd. Extract in Human Colon Cancer Cells
Source: Int J Mol Sci. 2020 Mar 19;21(6):2102. doi: 10.3390/ijms21062102 (PMC7139529; doi:10.3390/ijms21062102)
Supplement: Supplementary file 1 [file ijms-21-02102-s001.pdf]

Supporting Information

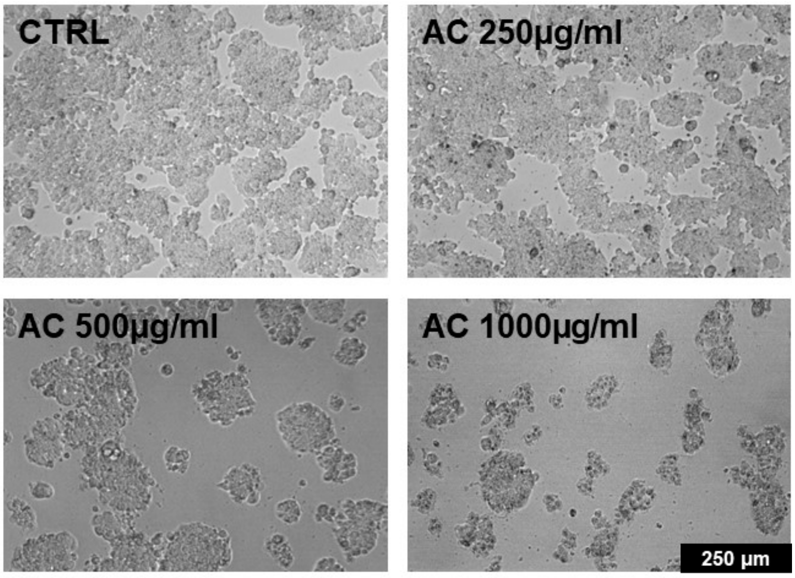

Figure S1. Effects of *Acacia catechu* Willd. extract (AC) for 24h on HT-29 cell viability. The cells were incubated with increasing concentrations of AC (250-1000 µg/ml) and morphological comparison performed at contrast phase microscopy (scale bar 250 µm). Each photograph was representative of three independent observations.

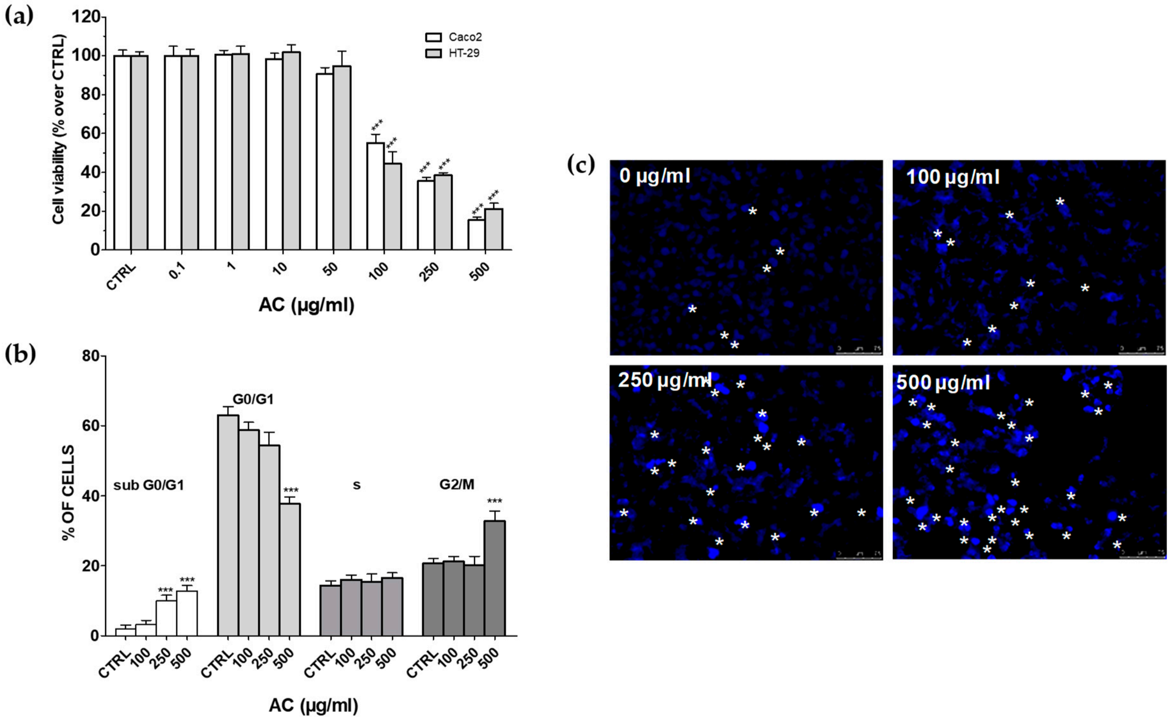

Figure S2 (**a**). Human colorectal adenocarcinoma Caco2 cells viability after treatment with *Acacia catechu* Willd. extract (AC) for 24h. The effects of the extract on HT-29 cells were also depicted from Figure 2 Panel A for comparison**.** (**b**). *Acacia catechu* Willd. extract (AC)-mediated effects on human colorectal adenocarcinoma Caco2 cells cycle. Percent of cells in subG0/G1, G0/G1, s and G2/M phase. In both panels, values are means ± S.E.M. of at least three independent experiments in which 4

points/concentration/time were run; controls (AC 0 μg/mL) represent untreated cells. Statistical significance was assessed by ANOVA followed by Dunnett post-test. (a): *** $p < 0.001$ vs controls. (**b**): *** $p < 0.001$ vs controls. (**c**). DNA condensation and damage assessed by DAPI staining in Caco-2 cells. Asterisks indicate cells with fragmented nuclei and condensed DNA, considered as apoptotic. Each photograph was representative of three independent observations (scale bar 75 μM).
